# Microglia in mouse retina contralateral to experimental glaucoma exhibit multiple signs of activation in all retinal layers

**DOI:** 10.1186/1742-2094-11-133

**Published:** 2014-07-26

**Authors:** Blanca Rojas, Beatriz I Gallego, Ana I Ramírez, Juan J Salazar, Rosa de Hoz, Francisco J Valiente-Soriano, Marcelino Avilés-Trigueros, Maria P Villegas-Perez, Manuel Vidal-Sanz, Alberto Triviño, José M Ramírez

**Affiliations:** 1 Instituto de Investigaciones Oftalmológicas Ramón Castroviejo, Facultad de Medicina, Pab VI, 4a, Avenida Complutense s/n, Universidad Complutense de Madrid, 28040 Madrid, Spain; 2 Departamento de Oftalmología y ORL, Facultad de Medicina, Avenida Complutense s/n, Universidad Complutense de Madrid, 28040 Madrid, Spain; 3 Facultad de Óptica y Optometría, Avda. Arcos de Jalón, 118, Universidad Complutense de Madrid, 28037 Madrid, Spain; 4 Experimental Ophthalmology Laboratory, Department of Ophthalmology, College of Medicine, Calle Campus Universitario s/n, University of Murcia, Regional Campus of International Excellence: Campus Mare Nostrum, Murcia Institute of Bio-Health Research (IMIB), E-30100 Murcia, Spain

**Keywords:** Microglia, Glaucoma, Retina, Mouse, Inflammation

## Abstract

**Background:**

Glaucomatous optic neuropathy, a leading cause of blindness, can progress despite control of intraocular pressure - currently the main risk factor and target for treatment. Glaucoma progression shares mechanisms with neurodegenerative disease, including microglia activation. In the present model of ocular hypertension (OHT), we have recently described morphological signs of retinal microglia activation and MHC-II upregulation in both the untreated contralateral eyes and OHT eyes. By using immunostaining, we sought to analyze and quantify additional signs of microglia activation and differences depending on the retinal layer.

**Methods:**

Two groups of adult Swiss mice were used: age-matched control (naïve, n = 12), and lasered (n = 12). In the lasered animals, both OHT eyes and contralateral eyes were analyzed. Retinal whole-mounts were immunostained with antibodies against Iba-1, MHC-II, CD68, CD86, and Ym1. The Iba-1+ cell number in the plexiform layers (PL) and the photoreceptor outer segment (OS), Iba-1+ arbor area in the PL, and area of the retina occupied by Iba-1+ cells in the nerve fiber layer-ganglion cell layer (NFL-GCL) were quantified.

**Results:**

The main findings in contralateral eyes and OHT eyes were: i) ameboid microglia in the NFL-GCL and OS; ii) the retraction of processes in all retinal layers; iii) a higher level of branching in PL and in the OS; iv) soma displacement to the nearest cell layers in the PL and OS; v) the reorientation of processes in the OS; vi) MHC-II upregulation in all retinal layers; vii) increased CD68 immunostaining; and viii) CD86 immunolabeling in ameboid cells. In comparison with the control group, a significant increase in the microglial number in the PL, OS, and in the area occupied by Iba-1+ cells in the NFL-GCL, and significant reduction of the arbor area in the PL. In addition, rounded Iba-1+ CD86+ cells in the NFL-GCL, OS and Ym1+ cells, and rod-like microglia in the NFL-GCL were restricted to OHT eyes.

**Conclusions:**

Several quantitative and qualitative signs of microglia activation are detected both in the contralateral and OHT eyes. Such activation extended beyond the GCL, involving all retinal layers. Differences between the two eyes could help to elucidate glaucoma pathophysiology.

## Background

Glaucoma is a disease in which intraocular pressure (IOP) has been traditionally considered the major risk factor [1,2]. Although it is known that IOP increase is not the only risk factor for glaucoma, it remains the main target in the treatment of glaucomatous neurodegeneration [3]. However, in some patients, optic-nerve degeneration reportedly progresses despite IOP control [4]. Recent studies indicate that glaucomatous disease can also be induced by an auto-immune response [5,6]. These observations suggest that we should consider glaucoma not as a disease involving raised pressure, but as a disease in which neurological sensitivity to pressure itself is independent of the magnitude [7]. Glaucoma is currently considered a neurodegenerative disease [8]. Taking into account that the retina and the optic nerve are projections of the central nervous system, it is not surprising that glaucoma could share mechanisms, such as neurodegeneration progression, with neurodegenerative diseases of diverse etiologies [9]. It has been reported that in glaucoma, disease progression occurs through mechanisms integrated in neuronal degenerative processes of compartmentalization [10], in which the microglia are a common cellular element that is activated.

Microglia are central nervous system resident innate immune cells, endowed with sensor and effector functions as well as with phagocytic capacity during physiological and pathological conditions [11,12]. These cells respond to neuronal stress or injury by adopting a so-called activated state, in which they progress from a resting mode to an activated phenotype [13,14]. The activated phenotype includes alterations in cellular morphology, changes in the structure of the cellular processes, tissue distribution, migratory characteristics, proliferation, expression of various growth factors and cytokines, or phagocytic activity.

Reactive microglia have been detected in the retina and optic nerve from axotomized eyes [15-23], after ischemia and reperfusion injury [24-27], in genetic or inducible ocular hypertension models [28-36], and in human glaucoma [37-39]. Microglial activation is thought to be a major contributor to neuronal death. The addition of minocycline (which inactivates microglia) has been shown to have neuroprotective effects, delaying retinal ganglion cell (RGC) death and axon loss in different models of glaucoma [40-42]. However, the mechanisms controlling microglial recruitment and activation in human glaucoma or animal models have not been established, and it is unclear when during the course of the disease the microglia undergo these changes [12].

In the mouse model of unilateral laser-induced ocular hypertension, morphological signs of activation and upregulation of major histocompatibility complex class II (MHC-II) have been reported in both treated and the normotensive untreated contralateral eyes [36]. However, more specific data concerning microglia activation in this model, and differences depending on the retinal layer remain unknown.

The aim of the present study is to analyze, in addition to variable morphology in the different layers of the retina, different signs of microglial activation both in contralateral eyes and in laser-induced OHT eyes, specifically: migration, microglia cell number, cell arbor area in the plexiform layers, area occupied by Iba-1+ cells in the NFL-GCL, and upregulation of activation markers (MHC-II, CD68, CD86, and Ym1).

## Materials and Methods

### Ethics statement

Mice were treated in accordance with Spanish law and the Guidelines for Humane Endpoints for Animals Used in Biomedical Research. This study was approved by the Ethics Committee for Animal Research of Murcia University and the Animal Health Service of the Murcia Regional Ministry of Agriculture and Water (approval ID number: A1310110807). In addition, animal procedures followed institutional guidelines, European Union regulations for the use of animals in research, and the Association for Research in Vision and Ophthalmology (ARVO) statement for the use of animals in ophthalmic and vision research.

### Animals and anesthetics

The experiments were performed on adult male albino Swiss mice (between 40 and 45 g) obtained from the breeding colony of the University of Murcia (Murcia, Spain). The animals were housed in temperature- and light-controlled rooms with a 12 hours light/dark cycle and *ad libitum* access to food and water. Light intensity within the cages ranged from 9 to 24 lux. All surgical procedures were performed under general anesthesia induced with an intraperitoneal (ip) injection of a mixture of ketamine (75 mg/kg, Ketolar®, Parke-Davies, Barcelona, Spain) and xylazine (10 mg/kg, Rompún®, Bayer, Barcelona, Spain). During recovery from anesthesia, the mice were placed in their cages and an ointment containing tobramycin (Tobrex®; Alcon, Barcelona, Spain) was applied to the cornea to prevent corneal desiccation and infection. Additional measures were taken to minimize discomfort and pain after surgery. The animals were killed with an ip overdose of pentobarbital (Dolethal Vetoquinol®, Especialidades Veterinarias, Alcobendas, Madrid, Spain).

### Experimental groups

Two groups of mice were considered for study: an age-matched control (naïve, n = 12) and a lasered group (n = 12) that was killed two weeks after lasering.

### Induction of ocular hypertension and IOP measurements

To induce OHT, the left eyes of anesthetized mice were treated in a single session with a series of diode laser (Viridis Ophthalmic Photocoagulator-532 nm, Quantel Medical, Clermont-Ferrand, France) burns, following previously described methods [43,44]. Briefly, the laser beam was directly delivered without any lenses, aimed at the limbal and episcleral veins. The spot size, duration, and power were between 50 and 100 μm, 0.5 seconds, and 0.3 W, respectively. Each eye received between 55 and 76 burns.

With the mice under deep anesthesia, the IOP was measured in both eyes with a rebound tonometer (Tono-Lab, Tiolat, Helsinki, Finland) [43,45-47] prior to and 24 hours, 48 hours, and 1 week after laser treatment for the lasered group, and before being killed for the naïve group. At each time point, six consecutive readings were taken for each eye and averaged. To avoid fluctuations of the IOP due to the circadian rhythm in albino Swiss mice [48], or due to the rise of the IOP itself [49], we tested the IOP consistently around the same time, preferentially in the morning and directly after deep anesthesia in all animals (lasered group and naïve).

### Immunohistochemistry

The mice were deeply anesthetized, perfused transcardially through the ascending aorta first with saline and then with 4% paraformaldehyde in 0.1 M phosphate buffer (PB) (pH 7.2 to 7.4). The orientation of each eye was carefully maintained with a suture placed on the superior pole immediately after deep anesthesia and before perfusion fixation [43]. Moreover, upon dissection of the eye, the insertion of the rectus muscle and the nasal caruncle were used as additional landmarks [50]. The eyes were post-fixed for two hours in the same fixative and kept in sterile 0.1 M PB. Retinas were then dissected and processed as retinal whole-mounts [51].

For the analysis of the microglia population in the mice retina and the expression of MHC class II molecules, retinal whole-mounts from naïve (n = 3) and OHT eyes (n = 3), and their contralateral eyes (n = 3), were double immunostained, as described elsewhere [36]. The following primary antibodies were used for immunostaining: rabbit anti-Iba-1 (Wako, Osaka, Japan) in a 1:500 dilution and rat anti-mouse MHC class II (I-A/I-E) (eBioscience; San Diego, California, United States) in a 1:100 dilution. Binding sites of the primary antibodies were visualized with the corresponding secondary antibodies: donkey anti-rabbit Alexa Fluor 594 (Invitrogen, Paisley, United Kingdom) in a 1:800 dilution and goat anti-rat Alexa Fluor 488 (Invitrogen, Paisley, United Kingdom) in a 1:150 dilution. For ease of reference, working dilutions for anti-Iba-1 and anti-MHC-II and their corresponding secondary antibody are omitted for simplicity.

For the study of the expression of CD68 (which recognizes a single-chain heavily glycosylated protein of 90 to 110 kD that is expressed on the lysosomal membrane of active phagocytic cells) in retinal microglia, retinas of naïve (n = 3) and OHT eyes (n = 3), and their contralateral eyes (n = 3) were double immunostained with anti-Iba1 and anti-CD68. The working dilution was 1:40 for CD68 rat anti-mouse (AbD Serotec, Oxford, United Kingdom). Binding sites of anti-CD68 were visualized after two days of incubation with the secondary antibody goat anti-rat Alexa Fluor 488 (Invitrogen, Paisley, United Kingdom) in a 1:150 dilution.

For the study of the expression of CD86 (which recognizes a co-stimulatory molecule) on retinal microglia, retinas of naïve (n = 3) and OHT eyes (n = 3), and their contralateral eyes (n = 3) were double immunostained with anti-Iba1 and anti-CD86. The working dilution was 1:25 for rat anti-mouse CD86 (BD Pharmigen Europe, Madrid, Spain). Binding sites of anti-CD86 were visualized after two days of incubation with the secondary antibody donkey anti-rat Alexa Fluor 488 (Invitrogen, Paisley, United Kingdom) in a 1:300 dilution.

For the study of the expression of Ym1 (which recognizes a protein from the lectin family synthesized and secreted by alternatively activated macrophages during inflammation) on retinal microglia, retinas of naïve (n = 3) and OHT eyes, (n = 3), and their contralateral eyes (n = 3) were double immunostained with anti-MHC class II (eBioscience; San Diego, California, United States) and anti-Ym1. The working dilution was 1:75 for rabbit anti-Ym1 (StemCell Technologies, Grenoble, France). Binding sites of anti-Ym1 were visualized after two days of incubation with the secondary antibody donkey anti-rabbit Alexa Fluor 594 (Invitrogen, Paisley, United Kingdom) in a 1:800 dilution.

In all instances, a negative control was performed to demonstrate that the secondary antibody reacted only with its respective primary antibody. This control was made by eliminating the primary antibody and replacing it with an antibody buffer. In addition to identifying the contribution of the endogenous fluorescence to the observed label, a tissue sample was incubated in all the buffers and detergents used in the experiment but without antibodies [51].

Retinas were analyzed and photographed with the ApoTome device (Carl Zeiss, Munich, Germany) and with a digital high-resolution camera (Cool- SNAP Photometrics, Tucson, Arizona, United States) coupled to a fluorescence microscope (Axioplan 2 Imaging Microscope Carl Zeiss, Munich, Germany). The microscope was equipped with appropriate filters for fluorescence-emission spectra of Alexa fluor 488 (Filter set 10, Zeiss), Alexa fluor 594 (Filter set 64, Zeiss) and DyLight 405 (Filter set 49, Zeiss). The ApoTome uses the ‘structured illumination’ method that enables conventional microscopy to create optical sections through the specimen and thereby improve the contrast and resolution along the optical axis. Z-stacks acquired under the ×63 objective were analyzed in Axiovision version 4.2 (Carl Zeiss, Munich, Germany) with Inside4D module in order to perform cut-view analysis. A cut-view is a software-generated reconstruction of the xz and yz planes of the z-stack, allowing visualization through the depth of the acquired z-stack.

Adobe Photoshop CS3 Extended 10.0 (Adobe Systems, Inc., San Jose, California, United States) was used for figure preparation.

### Retinal analysis

**Figure 1:**
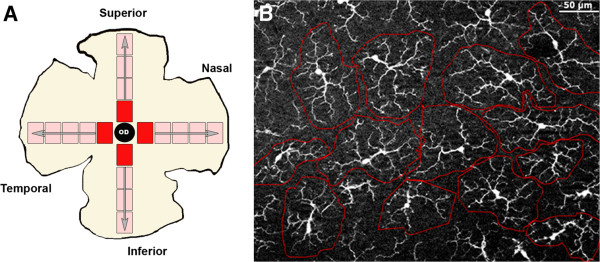
**Retinal whole-mount. A**: areas of retina selected for quantitative analysis of Iba-1+ cells. **B**: photomicrograph illustrating the method used for arbor-area quantification of Iba-1+ cells. A polygon was drawn manually by connecting the distal-most tips of the Iba-1+ cell processes.

To determine the effect of OHT on Iba-1+ cells, we quantified these cells in the retinal whole-mounts of naïve (n = 6), contralateral (n = 9), and OHT eyes (n = 9). Twenty-four equivalent areas of the retina were consistently selected for each retinal whole-mount in both the vertical and horizontal meridians which cross the optic nerve (Figure 1A). Each complete meridian selected in the retinal whole-mount was analyzed using the motorized stage of the microscope to scan their whole extension along the X-Y axis, respectively. Thus, all subsequent fields analyzed were contiguous and were examined systematically to ensure that no portion of the retinal whole-mount would be omitted or duplicated. Additionally, due to labeled Iba-1+ cells lying outside the immediate focal plane, we analyzed the whole preparation along the Z axis. These procedures were made at 20×, giving an area of 0.1502 mm^2^ per field analyzed.

The quantification method used depended on the cell number and cell-distribution characteristics of each retinal layer. The Interactive Measurement, a manual counting tool included in the AxioVision Release 4.8.2 computer program (Zeiss, Germany) in association with the ApoTome device coupled to the fluorescence microscope, was used for cell counting in the OS. Quantitative analysis of Iba-1+ somas was limited to those fully contained in the imaging space taken. For the plexiform layers and NFL-GCL, we created a reliable and quick algorithm of segmentation and control of distances developed in MATLAB (high-level technical computing language that can be used for image processing) [52]. In the IPL and OPL, Iba-1+ cells were distributed throughout the retina in a mosaic-like fashion without overlap between neighboring cells. This feature allows the algorithms to automatically determine the number of Iba-1+ cells. By contrast, in the NFL-GCL cell separation and distribution did not fulfill the criteria for automatic individual cell-counting, and therefore we quantified the area of the retina occupied by Iba-1+ cells (Iba1-RA) in this retinal layer [36,53]. For this purpose, images of the NFL-GCL were thus processed with a threshold tool in MATLAB. Thresholding defines a range of gray-scale values found on the pixels of objects of interest, differentiating them from other parts of the image based on the image’s gray scale. By using the pixel value information, we quantified the Iba1-RA in each photograph selected.

In a second step to further evaluate the effect of OHT in the Iba-1+ cell population, we analyzed in each eye the total arbor area of Iba-1+ cells (μm^2^) in four equivalent retinal areas of the selected ones used for cell-quantification analysis. The same analysis was performed in IPL and outer plexiform layer (OPL) due to the regular mosaic-like distribution of Iba-1+ cell in the plexiform layers in order to facilitate the measuring process. For this, we proceeded with a computer-assisted morphometric analysis. A polygon was drawn manually by connecting the distal-most tips of the Iba-1+ cell processes (Figure 1B) using the Interactive Measurement, tool of AxioVision (Zeiss, Germany), in association with the ApoTome device coupled to the fluorescence microscope.

### Statistical analysis

Data for the statistical analysis were introduced and processed in a SPSS 19.0 (comprehensive statistical software; SPSS Inc^©^, Armonk, New York, United States). Data are shown as mean ± SD. Statistical analyses were performed with *t*-test to identify differences among of the OHT, contralateral and naïve eyes as follows: i) IOP values; ii) Iba-1+ cell number in the photoreceptor outer segment (OS), OPL and IPL; iii) Iba1-RA in the NFL-GCL; and iv) arbor area of the Iba-1+ cells in the OPL and IPL. Differences were considered significant when *P* <0.05.

## Results

### Laser-induced ocular hypertension

The IOP values of OHT eyes (29.55 ± 4.44 mmHg) significantly differed from naïve values (16.16 ± 3.11 mmHg; *P* <0.001, one way analysis of variance (ANOVA) with Bonferroni method) and contralateral values (15.47 ± 1.57 mmHg; *P* <0.001, ANOVA with Bonferroni method). No significant differences were found between contralateral and naïve eyes.

#### Iba-1+ retinal cells: morphology and distribution

**Figure 2:**
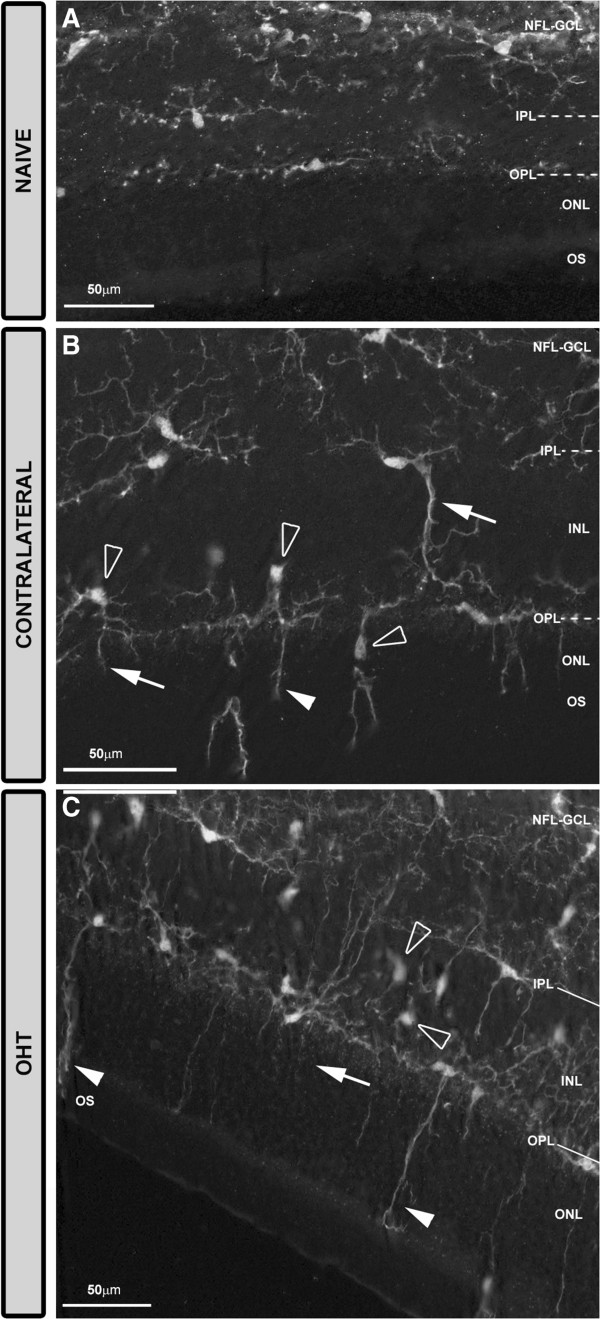
**Distribution of Iba-1+ cells in the retinal whole-mounts after 15 days of unilateral laser-induced OHT.** Iba-1 immunostaining. Retinal whole-mount. The pressure produced by the cover slip on the whole-mount caused a retinal-like section effect on one edge of the tissue that revealed that: in naïve eyes **(A)** Iba-1+ cells were distributed in the NFL-GCL, IPL and OPL. Somas and processes of ramified Iba-1+ cells ran parallel to the retinal surface. In contralateral eyes **(B)** somas (blank arrowhead) and processes (arrow) displaced to and extended to the INL and ONL, respectively. In the OPL the long processes of Iba-1+ cells reached the OS (arrowhead). In OHT eyes **(C)** the features observed in contralateral eyes **(B)** were more intense. (INL: inner nuclear layer; IPL inner plexiform layer; NFL-GCL: nerve fiber layer-ganglion cell layer; OHT: ocular hypertension; ONL: outer nuclear layer; OPL: outer plexiform layer; OS: photoreceptor outer segment).

**Figure 3:**
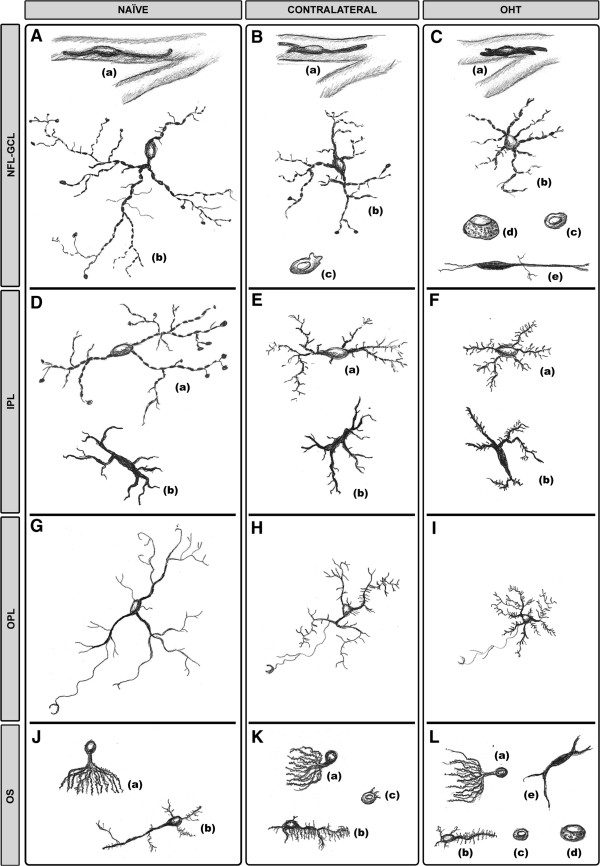
**Morphological changes in Iba-1+ cells.** The idealized schematic drawing illustrates the morphological types of Iba-1+ cells in the different retinal layers in naïve **(A, D, G, J),** contralateral **(B, E, H, K)** and OHT eyes **(C, F, I, L)**. Proportions between cell sizes were established taking into account the scale value of the microphotographs. In NFL-GCL **(A-C)**, both in contralateral **(B)** and in OHT eyes **(C),** perivascular cells (a) and ramified cells (b) showed a retraction of their cellular processes in comparison with naïve eyes **(A)**. Ameboid cells (c), absent from naïve eyes **(A)**, were observed in contralateral **(B)** and in OHT eyes **(C)**. Only in OHT eyes **(C)** were two additional morphological types of Iba-1+ cells observed: rounded cells (d) and rod-like cells (e). In the IPL **(D-F**) ramified cells (a) and dendritic-like cells (b) exhibited signs of activation (process retraction in (a) and increased secondary and higher-order processes (a, b)) in contralateral **(E)** and in OHT eyes **(F)**. In the OPL **(G-I)** ramified cell processes were retracted and were increased in contralateral **(H)** and in OHT eyes **(I)** in comparison with naïve eyes **(G)**. In the OS **(J-L),** cell orientation of type 1 OS Iba-1+ cells (a) changed from perpendicular in naïve eyes **(J)** to parallel to the retinal surface in contralateral **(K)** and in OHT eyes **(L)**. Processes of type 2 OS Iba-1+ cells (b) had a retraction of their processes and a hairy appearance in contralateral **(K)** and in OHT eyes **(L)**. Ameboid cells (c) were observed in contralateral **(K)** and OHT eyes **(L)** but not in naïve eyes **(J).** Only in OHT eyes **(L)** appeared rounded Iba-1+ cells (d) and cells with a dendritic-like appearance (e) in the OS. (NFL-GCL: nerve fiber layer-ganglion cell layer; IPL: inner plexiform layer; OHT: ocular hypertension; OPL: outer plexiform layer; OS: photoreceptor outer segment).

In the study groups, Iba-1+ cells were evenly distributed throughout the retina from the central zone to the periphery. They were distributed in different retinal layers: NFL-GCL, IPL, OPL, and OS (Figure 2).Most Iba-1+ cells within the naïve retina had a ramified morphology. Overall, they had a small ovoid or triangular soma from which three to four primary processes sprouted and then divided dichotomously into secondary ones. Ramified Iba-1+ cells were examined in the NFL-GCL, IPL, and OPL and their somas and processes ran parallel to the retinal surface. In the plexiform layers, ramified Iba-1+ cells were distributed throughout the retina in a mosaic-like fashion without overlapping between neighboring cells. Specific features of the ramified Iba-1+ cells as well as the presence of other morphological types varied depending on the retinal layer analyzed and on the study groups (naïve, contralateral, and OHT eyes) as shown in Figure 3.

#### Iba-1+ cells in the NFL-GCL

**Figure 4:**
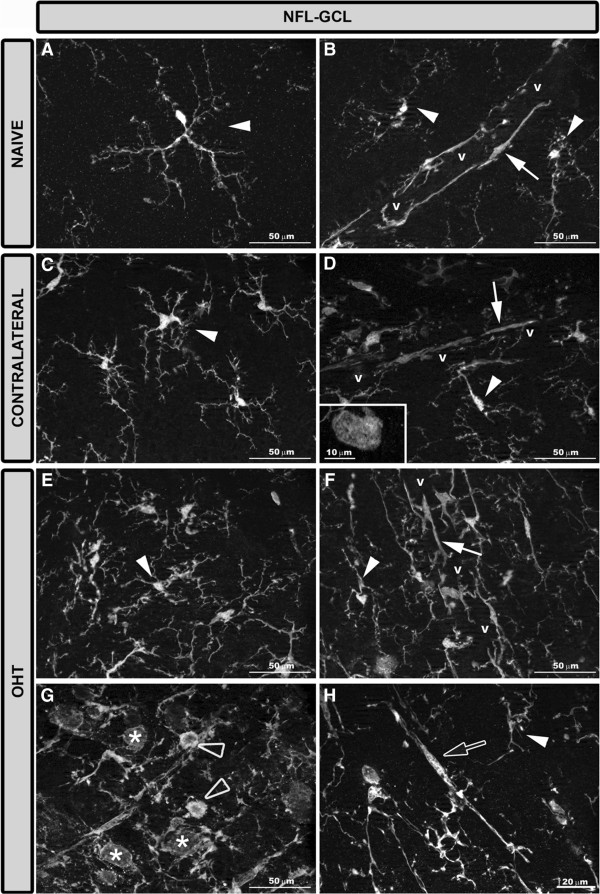
**Iba-1+ cells in the NFL-GCL after 15 days of unilateral laser-induced OHT.** Iba-1 immunostaining. Retinal whole-mount. In naive eyes **(A, B)** there were ramified Iba-1+ cells (arrowhead) with varicose processes and perivascular Iba-1+ cells (arrow) with elongated morphology and thick processes. Both in contralateral eyes **(C, D)** and in OHT eyes **(E-H)** ramified Iba-1+ cells (arrowhead in C-F, H) and perivascular Iba-1+ cells (arrow in D, F) showed a retraction of the cellular processes. Scarce ameboid Iba-1+ cells were detected in contralateral eyes (D inset) and more frequently in OHT eyes (blank arrowhead in G). Only in OHT eyes were two additional morphological types of Iba-1+ cells discerned **(G, H)**: rounded cells (asterisk in G) and rod-like cells with elongated cell bodies and two processes prominently projecting from each pole (blank arrow in **H**). (NFL-GCL: nerve fiber layer-ganglion cell layer; OHT: ocular hypertension; v: retinal vessel).

**Figure 5:**
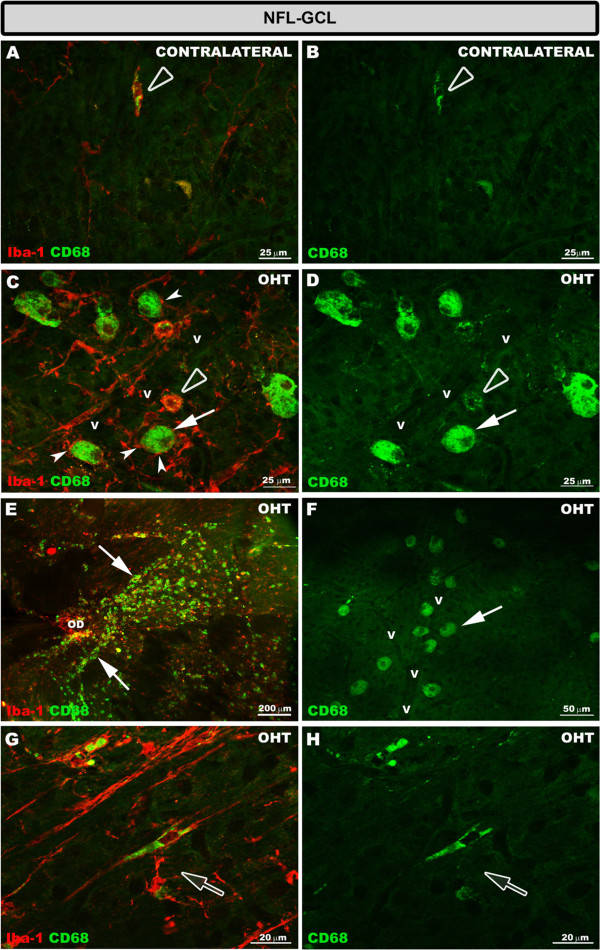
**CD68 expression in the NFL-GCL after 15 days of unilateral laser-induced OHT.** Double immunostaining: Iba-1/CD68. Retinal whole-mount. In contralateral eyes **(A, B)** CD68 immunoreaction was detected only in some ameboid Iba-1+ cells which exhibited a patchy staining pattern (blank arrowhead). In the retinal whole-mount of OHT eyes **(C-H),** the greater CD68 immunoreaction was observed in this retinal layer. This was because, in addition to the CD68 immunoreactivity of ameboid Iba-1+ cells (blank arrowhead in **C, D**), rounded Iba-1+ cells (arrow in **C-F**) and some rod-like cells (blank arrow in **G, H**) had intense CD68+ cytoplasmic staining. Rounded Iba-1+ CD68+ cells were adjacent to the retinal vessels **(C, D, F)**, being located mainly close to the optic disc **(E)** and in the periphery of the retina. In some instances, the processes of ramified Iba-1+ cell surrounded the rounded Iba-1+ cells (arrowhead in **C**) (NFL-GCL: nerve fiber layer-ganglion cell layer; OD: optic disc; OHT: ocular hypertension; v: retinal blood vessel).

In naïve eyes, two morphological types of Iba-1+ cells were observed in the NFL-GCL: ramified (Figures 3A, 4A,B) and perivascular (Figures 3A, 4B). In both instances, they are related to the blood vessels. Most Iba-1+ cells had a ramified morphology, varicose processes, and somas located on the retinal vessels and in the intervascular space. From both primary and secondary processes sprouted thin processes that on occasions ended as bulbous-tips (Figures 3A, 4A). Ramified Iba-1+ cells ran parallel to the retinal surface and in some instances, processes penetrating the layer perpendicularly were observed. Perivascular Iba-1+ cells showed elongated morphology and thick somas and processes. These cells were found in the vessel walls (Figures 3A, 4B), specifically on the surface of the retinal large vessels, in the vicinity of the optic nerve, and in the collecting tube venule of the peripheral retina.In contralateral eyes and OHT eyes the two morphological types of Iba-1+ cells described above showed a retraction of the cellular processes in comparison with naïve eyes (Figures 3B,C, 4C-H). In addition, scarce Iba-1+ cells with an ameboid morphology were observed in this layer in contralateral eyes (Figures 3B, 4D inset) and were more frequently found in OHT eyes (Figures 3C, 4G). Notably, two additional morphological types of Iba-1+ cells were observed only in OHT eyes: i) rounded cells (Figures 3C, 4G) and ii) rod-like cells (Figures 3C, 4H). Rounded cells were found adjacent to the major retinal vessels (Figure 5F) or in the neural parenchyma and were observed mainly in the vicinity of the optic disk (Figure 5E) and in the periphery of the retina. In some instances, the processes of ramified Iba-1+ cells surrounded the rounded Iba-1+ cells (Figure 5C). These cells showed a less intense Iba-1+ immunolabeling than did ameboid cells (Figure 4G). Rod-like cells had elongated cell bodies and two processes prominently projected from each pole which were aligned end-to-end, coupling to form trains associated with axons and not related to retinal vessels (Figure 4H).

#### Iba-1+ cells in the IPL

**Figure 6:**
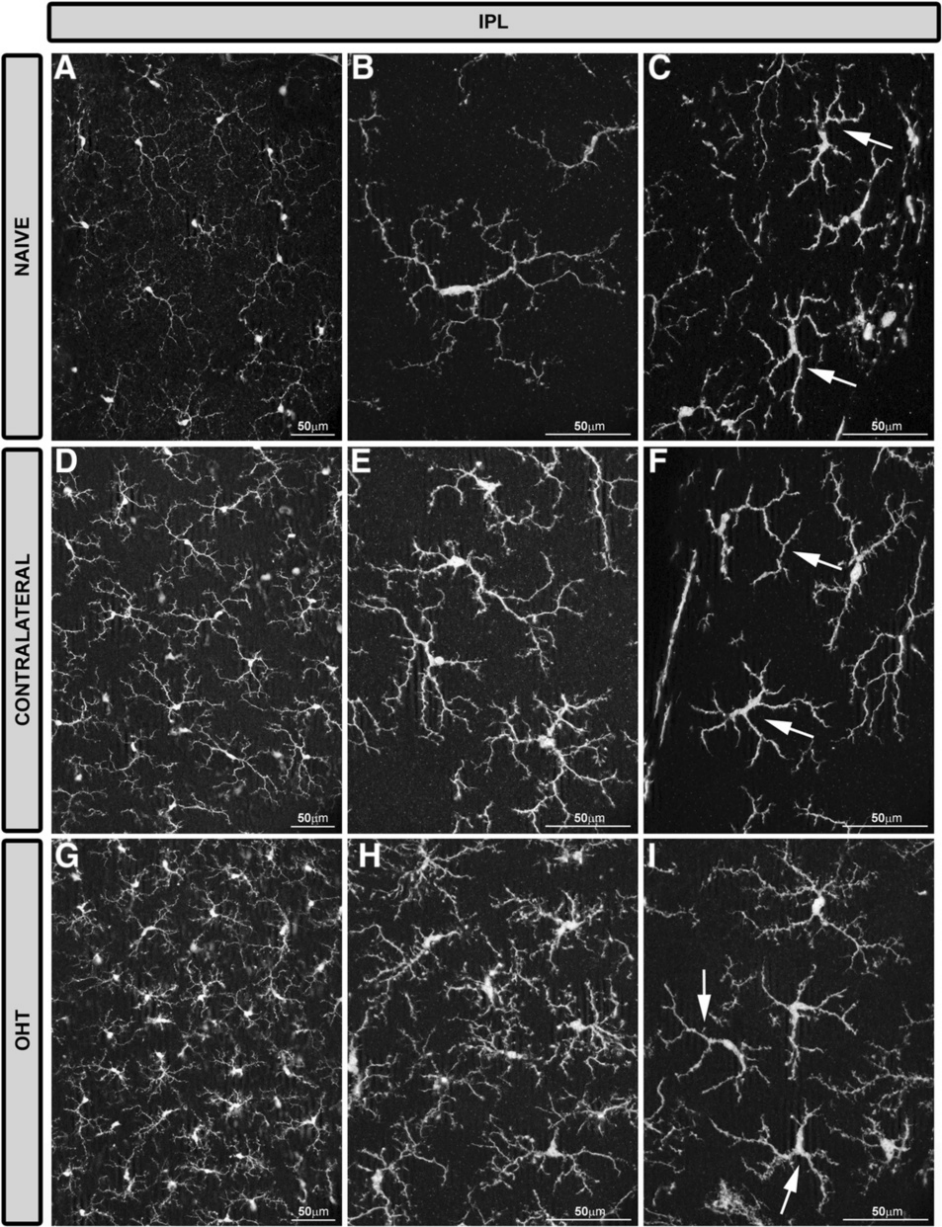
**Iba-1+ cells in the IPL after 15 days of unilateral laser-induced OHT.** Iba-1 immunostaining. Retinal whole-mounts. Most Iba-1+ cells in the IPL were ramified and there were few Iba-1+ cells with a dendritic-like appearance (arrow in **C, F, I**). In comparison with naïve eyes **(A-C)**, ramified Iba-1+ cells in the IPL of contralateral eyes and OHT eyes formed a denser cellular mosaic **(D, E, G, H)**. Both ramified and dendritic-like Iba-1+ cells exhibited morphological signs of cell activation (process retraction and increased secondary and superior order processes) that were more pronounced in OHT eyes **(G-I)** than in contralateral eyes **(D-F)**. (IPL: inner plexiform layer; OHT: ocular hypertension).

In the IPL of naïve eyes, two morphological types of Iba-1+ cells were distinguished: ramified and dendritic-like cells (Figures 3D, 6B).Most Iba-1+ cells in the IPL were ramified and their somas were located next to the GCL or inner nuclear layer (INL). In comparison with ramified cells in the NFL-GCL, they had fewer thin processes emerging from primary and secondary processes, which gave them a less ramified appearance (Figures 3D, 6B). Processes were varicose and most of them ended as bulbous-tips (Figures 3D, 6B). Some processes reached both the GCL and INL. In addition to the cells described above, a few Iba-1+ cells had thick somas and two to four thick primary processes and short, thick secondary ones, giving the cell a dendritic-like appearance (Figures 3D, 6C). Dendritic-like cells ran parallel to the retinal surface and were located in the juxtapapillary area and in the vicinity of the collecting tube venule in the peripheral retina.In comparison with naïve eyes, ramified Iba-1+ cells in the IPL of contralateral eyes and OHT eyes formed a denser cellular mosaic (Figure 6A,D,G) and exhibited: i) a retraction of the cellular processes (Figures 3E,F, 6E, H); ii) a disappearance of the bulbous-tip (Figures 3E,F, 6E,H); iii) numerous thin and short secondary and superior order processes which gave the cells a fuzzy appearance; iv) displacement of some somas into the INL and GCL (Figure 2B,C); and v) numerous perpendicular processes that penetrated adjoining layers (Figure 2B,C). Dendritic-like Iba-1+ cells in contralateral and OHT eyes had numerous thin and short secondary and superior order processes (Figures 3E,F, 6F,I). In both cellular types, the aforementioned signs of cell activation were more pronounced in OHT eyes (Figures 3F, 6G,H,I) than in contralateral eyes (Figures 3E, 6D,E,F).

#### Iba-1+ retinal cells in the OPL

**Figure 7:**
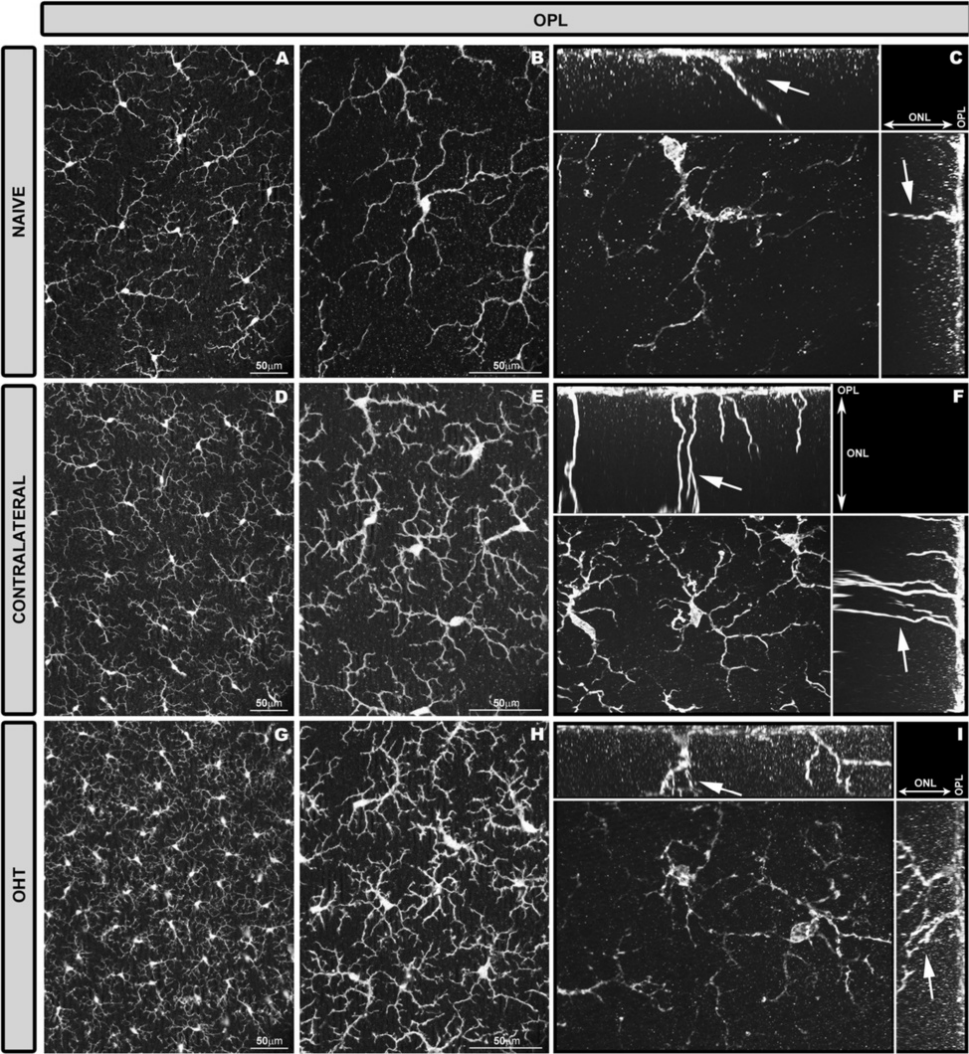
**Iba-1+ cells in the OPL after 15 days of unilateral laser-induced OHT.** Iba-1 immunostaining. Retinal whole-mount. In the OPL only ramified Iba-1+ cells were observed. In comparison with naïve eyes **(A-C)**, ramified Iba-1+ cells in the OPL of contralateral and OHT eyes formed a denser cellular mosaic **(D, E, G, H)**. Iba-1+ cells in this layer exhibited morphological signs of cell activation (process retraction and increased secondary and superior order processes) that were more pronounced in OHT eyes **(G-I)** than in contralateral eyes **(D-F)**. The cut-view analysis in the YZ (sagittal) and XY (coronal) plane demonstrate long processes (arrow) extending from the soma and running across the ONL into the OS **(C, F, I)**. These long processes were more frequently observed in contralateral **(F)** and OHT eyes **(I)** than in naïve eyes **(C)**. (OHT: ocular hypertension; ONL: Outer nuclear layer; OPL: outer plexiform layer, OS photoreceptor outer segment).

In the OPL of naïve, contralateral, and OHT eyes, only ramified Iba-1+ cells were detected. These formed a mosaic-like pattern denser than in the IPL (Figure 7).In naïve eyes (Figures 3G, 7A-C), a specific feature of these cells was the presence of numerous thin processes sprouting from the soma and the primary and secondary processes. Some processes extended to the INL and ONL. On occasions, a long process extending from the soma ran across the ONL into the OS (Figure 7C) where it ended in a goblet-like shape (Figure 3G).In contralateral and OHT eyes as compared with naïve eyes, ramified Iba-1+ cells in the OPL showed: i) retraction of processes (Figures 3H,I,7D,E,G,H); ii) numerous thin and short secondary and superior order processes (Figures 3H,I, 7E,F,H,I); iii) a displacement of the some somas to the INL and ONL (Figure 2B,C); iv) greater number of processes reaching the OS (Figure 7F,I) and; v) a denser cellular mosaic (Figure 7D,E,G,H). All these characteristics were more pronounced in the OHT eyes (Figures 3I, 7G-I) than in the contralateral eyes (Figures 3H, 7D-F).

#### Iba-1+ retinal cells in the OS

**Figure 8:**
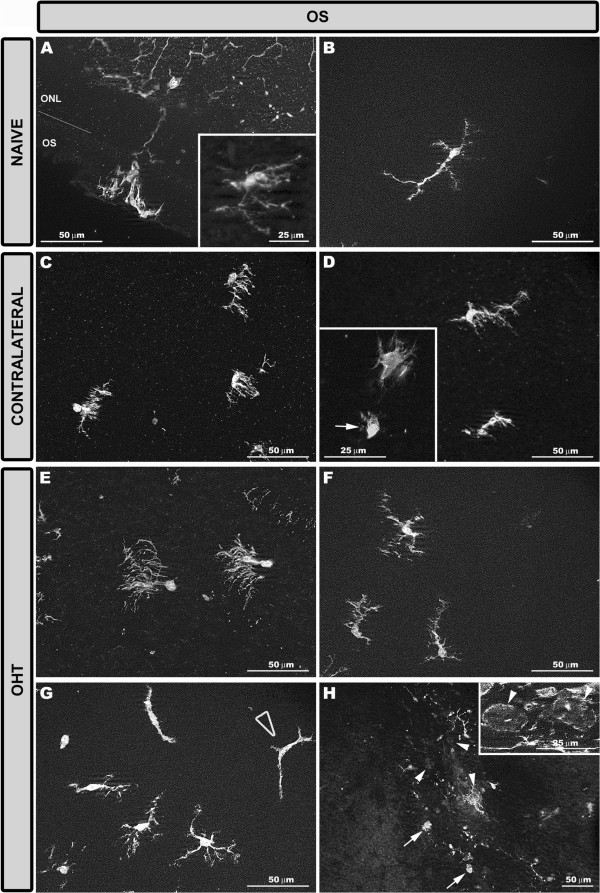
**Iba-1+ cells in the OS after 15 days of unilateral laser-induced OHT.** Iba-1 immunostaining. Retinal whole-mount. Type 1 **(A, C, E)** and type 2 **(B, D, F, G)** Iba-1+ cells in the OS. The retinal-like section effect produced by the pressure exerted by the cover slip on one edge of the tissue **(A)** allowed us to observe that in type 1 OS Iba-1+ cells numerous processes emerged from the same point of the soma, like the roots of a tree. In naïve eyes these processes extended across the thickness of the OS perpendicularly to the retinal surface. Consequently, soma and processes of type 1 OS cells were located on a different focal plane and therefore both cell structures could not be focused simultaneously during whole-mount analysis (**A** inset). In contralateral **(C)** and OHT eyes **(E)** type 1 OS Iba-1+ cells orientation changed from perpendicular to parallel to the retinal surface. In contralateral **(D)** and OHT eyes **(F)** type 2 OS Iba-1+ cells had a retraction of their processes and a hairy appearance. Ameboid Iba-1+ cells were observed in contralateral (**D** inset, arrow) and OHT eyes (arrow in **H**). Only in OHT eyes were two additional morphological types of Iba-1+ cells observed **(G, H)**: cells with a dendritic-like appearance (blank arrowhead in G) and rounded Iba-1+ cells (arrowhead in **H** and inset). (OHT: ocular hypertension; ONL: outer nuclear layer; OS: photoreceptor outer segment).

In three groups of eyes studied, Iba-1+ cells in the OS were unevenly distributed.In the naïve eyes, two main morphological types of Iba-1+ cells were observed depending on their morphology and location: i) cells with ovoid somas located near or inside the ONL and numerous processes emerging from the same point of the soma, like the roots of a tree (Type 1- OS) (Figures 3J, 8A). These processes extended across the thickness of the OS, perpendicularly to the retinal surface (Figure 8A). This morphological type was the predominant one in the OS; and ii) cells with ovoid somas located near the retinal pigment epithelium (RPE) and one thick primary process running parallel to the retinal surface. Sparse, thin, and short processes sprouted from the soma and the primary process (Type 2- OS) (Figures 3J, 8B). These cells were more frequently detected in the vicinity of the ora serrate.In comparison with naïve eyes, the morphology and arrangement of the two cell types described above varied in contralateral and OHT eyes. Type 1 OS Iba-1+ cells were displaced to the vicinity of RPE and formed groups that were randomly distributed throughout the layer. All the cells in a group had their processes oriented in the same direction (Figure 8C,E). In contralateral eyes, the orientation of the processes began to change from perpendicular to parallel to the retinal surface (Figures 3K, 8C). In OHT eyes, both the somas and the processes were clearly arranged parallel to the retinal surface (Figures 3L, 8E). In contralateral and OHT eyes the processes of type 2 OS Iba-1+ cells had a retraction of their processes and a hairy appearance due to abundant thin and short processes that sprouted from the soma and main processes (Figures 3K,L, 8D,F).As in the NFL-GCL, ameboid Iba-1+ cells were detected in contralateral (Figures 3K, 8D inset) and OHT eyes (Figures 3L, 8H) but were more frequently found in the OHT eyes. Also, two more morphological types of Iba-1+ cells were observed only in OHT eyes: i) cells with a dendritic-like appearance (Figures 3L, 8G), similar to IPL and, ii) rounded Iba-1+ cells (Figures 3L, 8H), which were scarce, located in the retinal periphery and showing a patchy distribution.

### Activation markers in Iba-1+ retinal cells: MHC-II, CD68, CD86, and Ym1

#### Age-matched control (naive)

**Figure 9:**
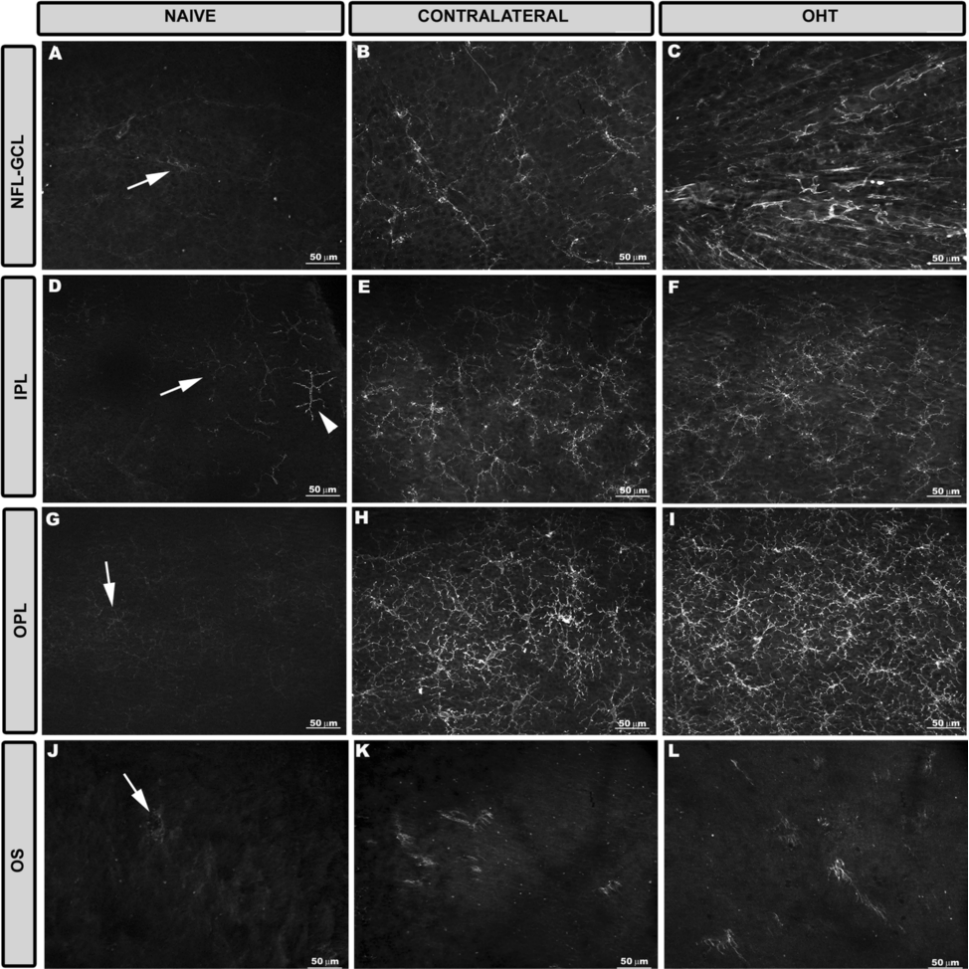
**MCH-II expression in the retinal layers after 15 days of unilateral laser-induced OHT.** MHC-II immunostaining. Retinal whole-mounts. In naïve eyes **(A, D, G, J)**, Iba-1+ cells had a weak constitutive MHC-II expression (arrow) except for the dendritic-like cells in the IPL which had an intense constitutive MHC-II expression (arrowhead in D). In contralateral **(B, E, H, K)** and OHT eyes **(C, F, I, L)** MHC-II was upregulated in all retinal layers. (NFL-GCL: nerve fiber layer-ganglion cell layer; IPL: inner plexiform layer; OHT: ocular hypertension; OPL: outer plexiform layer; OS: photoreceptor outer segment).

**Figure 10:**
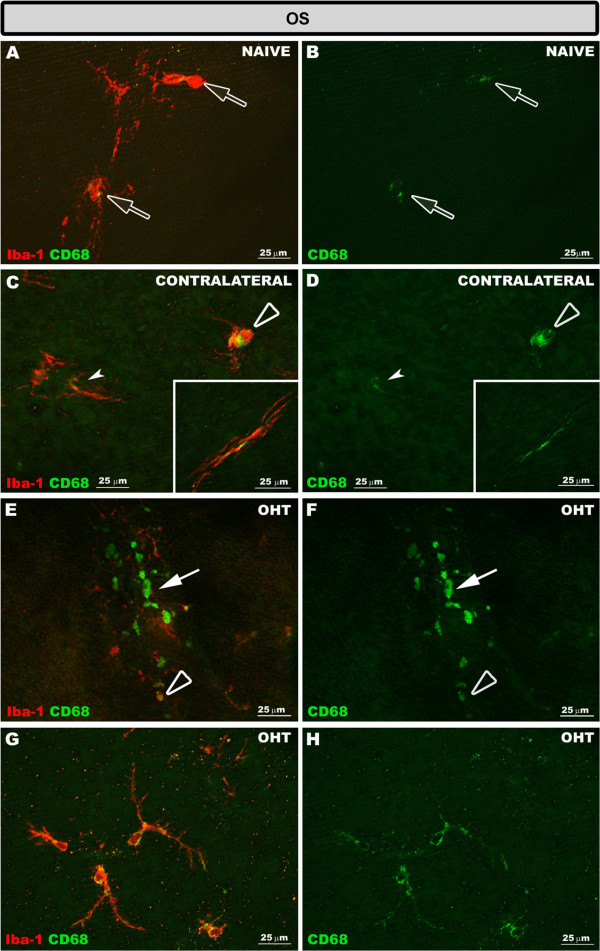
**CD68 expression in the OS after 15 days of unilateral laser-induced OHT.** Double immunostaining: Iba-1/CD68. Retinal whole-mount. In naïve eyes **(A, B)** CD68 immunostaining was observed in type 1 OS Iba-1+ cells (blank arrow). In the OS in contralateral eyes **(C, D)** ameboid Iba-1+ cells (blank arrowhead) had a greater CD68 immunostaining than type 1 OS (arrowhead) and type 2 OS (inset) Iba-1+ cells. In OHT eyes **(E-H)** CD68 immunostaining in the OS was observed in ameboid Iba-1+ cells (blank arrowhead in **E, F**), rounded Iba-1+ cells (arrow in **E, F**) and in the soma and processes of type 2 OS Iba-1+ cells **(G, H)**. (OHT: ocular hypertension; OS: photoreceptor outer segment).

Overall, Iba-1+ cells in naïve retinas had a weak constitutive MHC-II expression (Figure 9A,D,G,J; Additional file 1) except for the dendritic-like cells in the IPL, which had an intense constitutive MHC-II expression (Figure 9D). With respect to CD68, the immunostaining varied depending on the retinal layer analyzed. Thus, a punctate CD68 immunostaining was scarcely found in the soma of few Iba-1+ cells in the NFL-GCL and plexiform layers (Additional file 1). However, in the OS patches of CD68, immunostaining was found in most type 1 OS cells but only in some type 2 OS cells (Figure 10A,B; Additional file 1). No immunostaining for CD86 or Ym1 was found in naïve eyes (Additional file 1).

#### Contralateral and OHT eyes

**Figure 11:**
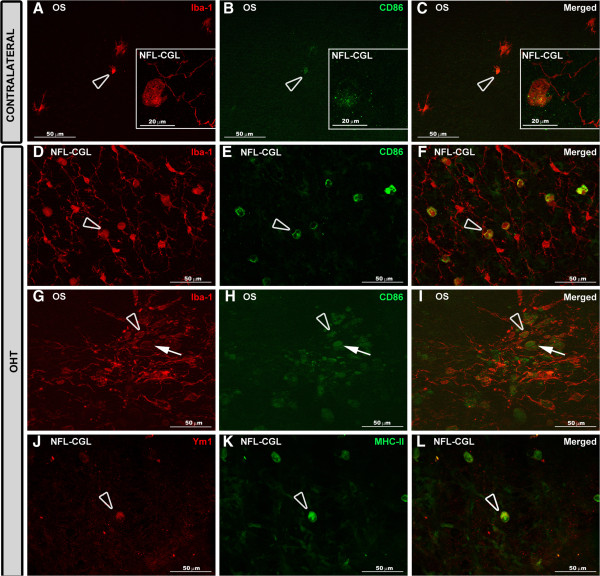
**CD86 and Ym1 expression in the retina after 15 days of unilateral laser-induced OHT.** Double immunostaining: Iba-1/CD86 **(A-I)** and MHC-II/Ym1 **(J-L)**. Retinal whole-mount. In contralateral eyes **(A-C)** CD86 immunostaining was observed in some ameboid Iba-1+ cells in the OS (blank arrowhead) and NFL-GCL (inset). In OHT eyes **(D-I)** rounded Iba-1+ cells (arrow) and most ameboid Iba-1+ cells (blank arrowhead) were CD86+. Ym1 immunoreaction **(J-L)** was restricted to a few ameboid Iba-1+ cells (blank arrowhead) in the NFL-GCL of OHT eyes. (NFL-GCL: nerve fiber layer-ganglion cell layer; OHT: ocular hypertension; OS: photoreceptor outer segment).

Both in contralateral (Figure 9B,E,H,K) and OHT eyes (Figure 9C,F,I,L), Iba-1+ cells showed upregulation in MHC-II expression in all retinal layers (Additional file 1). In contralateral eyes CD68 immunostaining was similar to naïve eyes except for a few ramified Iba-1+ cells in the IPL which had a patchy CD68+ immunostaining instead of the punctate immunostaining observed in naïve eyes (Additional file 1). In addition, ameboid Iba-1+ cells in the NFL-GCL (Figure 5A,B) and OS (Figure 10C,D) in contralateral eyes exhibited intense CD68 immunostaining. CD86 immunostaining was restricted to some ameboid Iba-1+ cells (Figure 11B,C; Additional file 1). No Ym1+ cells were observed (Additional file 1).

By contrast, in OHT eyes most Iba-1+ cells had CD68 immunostaining in the somas and in some processes that varied from punctate to patchy (Figure 10G,H; Additional file 1). The retinal layer having the greatest CD68 immunostaining was the NFL-GCL and OS, due to ameboid cells (Figure 10E,F, 5C,D), and the intense CD68+ cytoplasm exhibited by rounded Iba-1+ cells (Figures 10E, F, 5C,D,E,F) and some rod-like microglia (Figure 5G,H, Additional file 1). CD68 cytoplasmic staining in rounded Iba-1+ cells had a vacuolated appearance (Figure 5C,D). CD86 immunostaining was detected in ameboid Iba-1+ cells (Figure 11E,F,H,I) and rounded Iba-1+ cells (Figure 11H,I). Ym1 was restricted to few ameboid MHC-II cells in the NFL-GCL (Figure 11K,L).

### Quantitative analysis of Iba-1+ retinal microglial cells

#### Number of Iba-1+ cells in the IPL, OPL, and OS

**Figure 12:**
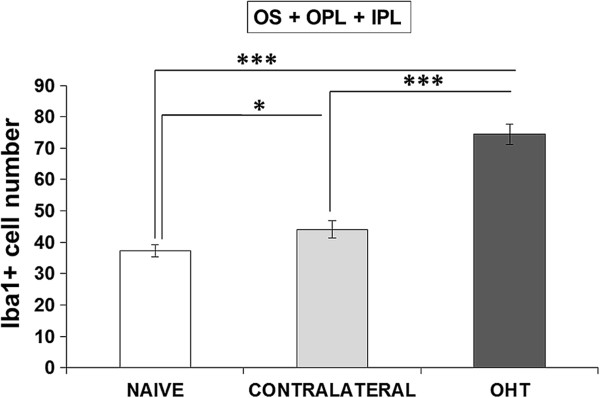
**Iba-1+ cell quantification in OS, OPL, and IPL.** Each bar represents the mean ± SD of the sum of Iba-1+ cell number contained in the three retinal layers. ***P <0.001 versus naïve and contralateral retinas. *P <0.05 versus naïve retinas. (IPL: inner plexiform layer; OHT: ocular hypertension; OPL: outer plexiform layer; OS: photoreceptor outer segment).

OHT eyes had significantly more Iba-1+ cells in the IPL, OPL, and OS than did contralateral and naïve eyes, both when the comparison was made as the sum of Iba-1+ cells contained in the three layers (Iba-1+ total number, Figure 12) or when the layers were compared one by one between the study groups (Table 1) (*P* <0.001 in all instances; *t-*test). The Iba-1+ total number also significantly increased in contralateral eyes in comparison with naïve eyes (*P* <0.05; unpaired *t-*test; Figure 12). The analysis by layers revealed that the IPL of contralateral eyes had significantly more Iba-1+ cells than did naïve eyes (*P* <0.05; unpaired *t*-test) (Table 1). In addition, the comparison between OPL and IPL showed that the number of Iba-1+ cells was significantly greater in the OPL in naïve (*P* <0.01), contralateral (*P* <0.001) and OHT eyes (*P* <0.001; paired *t-*test in all instances; Table 1).

**Table 1:** Iba-1+ cell quantification by retinal layers

	**OS**	**OPL**	**IPL**	**OS + OPL + IPL**
NAIVE	1.44 ± 0.38	20.00 ± 1.16	15.87 ± 0.87	37.31 ± 1.84
CONTRALATERAL	3.35 ± 1.09	22.36 ± 1.28	18.43 ± 1.26	44.14 ± 2.67
OHT	8.80 ± 1.43	34.81 ± 1.41	30.80 ± 1.59	74.42 ± 3.27

Data are presented as the mean number of Iba-1+ cells ± SD. Measurements were made at 20×, giving up an area of 0.1502 mm^2^ per field analyzed. (IPL: inner plexiform layer; OHT: ocular hypertension; OPL: outer plexiform layer; OS: photoreceptor outer segment).

#### Area of the retina occupied by Iba-1+ cells (Iba1-RA) in the NFL-GCL

**Figure 13:**
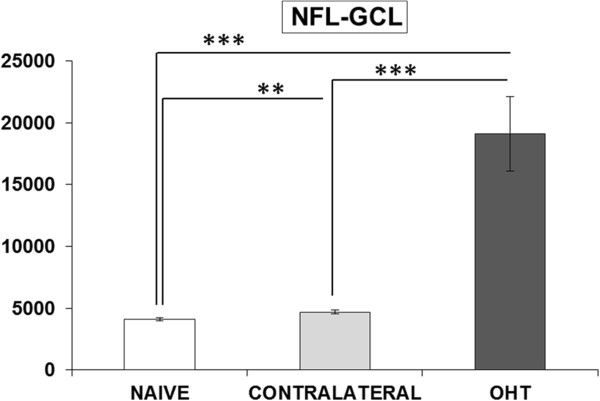
**Area of the retina occupied by Iba-1+ cells in the NFL-GCL.** Each bar represents the mean ± SD of area of the retina occupied by Iba-1+ cells. ***P <0.001 versus naïve and contralateral retinas. **P <0.01 versus naïve retinas. (NFL-GCL: nerve fiber layer-ganglion cell layer; OHT: ocular hypertension; RA: retinal area) (y axis: Iba1-RA (μm^2^)).

In the NFL-GCL, the area of the retina occupied by Iba-1 (+) cells (Iba1-RA) in OHT eyes (19090.57 ± 6040.56) significantly increased in comparison both to contralateral (4689.97 ± 359.47; *P* <0.001; paired *t-*test) and to naïve eyes (4096.94 ± 260.97; *P* <0.001; unpaired *t-*test; Figure 13). Notably, Iba1-RA in contralateral eyes was significantly higher than in naïve eyes (*P* <0.01; unpaired *t-*test; Figure 13).

#### Quantification of the arbor area of the Iba-1+ cells

**Figure 14:**
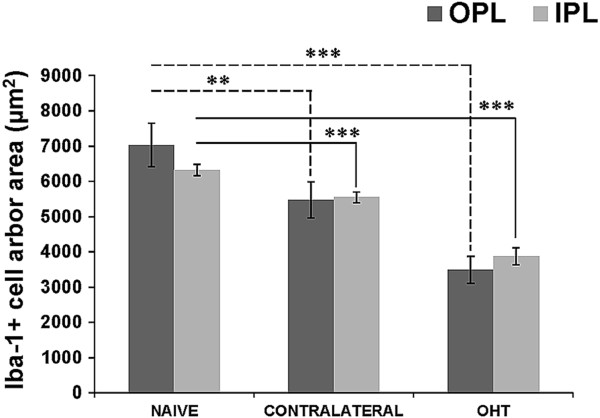
**Arbor area of Iba 1+ cells in the plexiform layers.** Each bar represents the mean ± SD of arbor area of the Iba-1+ cells. Dotted lines represent comparison among OPL values: ***P <0.001 versus OHT retinas; **P <0.01 versus contralateral retinas. Solid lines represent comparison among IPL values: ***P <0.001 versus contralateral and OHT retinas. (OHT: ocular hypertension; IPL: inner plexiform layer; OPL: outer plexiform layer; OS photoreceptor outer segment).

The average arbor area of the Iba-1+ cells in the IPL and the OPL was significantly reduced in OHT eyes (3492.75 ± 766.85 for IPL and 3868.80 ± 477.14 for OPL) compared to both contralateral (5473.82 ± 1023.67 for IPL and 5545.96 ± 288.80 for OPL) and naïve eyes (7031.81 ± 1238.39 for IPL and 6318.65 ± 319.17 for OPL) and in contralateral eyes compared to naïve eyes (Figure 14).

## Discussion

It is currently known that reactive microgliosis associated with retinal damage takes place both in human glaucoma [37-39] as well as in experimental models of OHT [28-34,54]. In the experimental model of OHT used in the present work, we have previously reported that 15 days after laser treatment: i) the microglia of OHT eyes and contralateral untreated eyes had signs of activation [36], and ii) such activation is not observed in eyes receiving laser in the non-draining portion of the sclera (avoiding the aqueous-collecting system) in which IOP values did not differ from those of the naïve group [36]. However, more specific data concerning microglia activation in this model and differences depending on the retinal layer remain unknown. Therefore, in the present study we show a detailed description of multiple signs of activation displayed by the retinal microglia in the different layers of the retina, specifically: morphological changes, cell displacement, increased cell number, upregulation of activation markers (MHC-II, CD68, CD86, and Ym1) and quantification of the area occupied by Iba-1+ cells in the NFL-GCL and the arbor area of Iba-1+ cells in the plexiform layers. Our results confirm that 15 days after lasering, the microglia in all retinal layers underwent multiple changes, both in the lasered eye as well as in the contralateral untreated eye.

In the model of laser-induced OHT used in the present work, a substantial increase in the IOP was evident 24 hours after the lasering of the limbal and episcleral veins. This continued for four days and then gradually returned to the basal value after the fifth day, so that one week after lasering, the IOP values in the treated animals were comparable for both eyes [43].

Previous reports on the experimental model of OHT used here showed abnormal electroretinograms [43,44], indicating that cellular involvement extends beyond RGC, supporting findings reported in human glaucoma [55] and in other experimental models of OHT [56-59]. According to this, we observed microglial reactivity in all retinal layers of OHT and contralateral eyes.

In the normal adult nervous system, microglia are found in a quiescent state characterized by a ramified morphology [60]. When microglia detects an insult, the cell becomes activated (shortening and widening of microglial processes) and can transform into a macrophage-like morphology known as ameboid microglia defined by the absence of the cell processes [14,61-64]. A process of shortening in Iba-1+ cells was noted in all retinal layers in contralateral and OHT eyes and could be consistent with the significant reduction of the Iba-1+ cell-arbor area found in the plexiform layers. Ameboid microglia has been reported in acute processes and neurodegenerative diseases such as Alzheimer’s disease and multiple sclerosis [65]. Ameboid microglia is commonly found in the vicinity of lesions in neuroinflammatory disorders [66]. According to this scenario, ameboid CD68+ Iba-1+ cells were found in OHT eyes as well as in contralateral eyes where RGC death has not been reported [43].

In addition to the classical signs of microglial reactivation mentioned above, hyper-ramified microglia, an intermediate activation stage between the resting and reactive forms, has been described [67]. Hyper-ramified microglia have been seen both in the presence of several non-pathological tissue signals [68-70], as well as in cerebral degenerative processes related to axonal damage [71-73]. In the present study, Iba-1+ cells with raised levels of branching were observed in contralateral and OHT eyes in all retinal layers except in the NFL-GCL. In the plexiform layers of OHT eyes, these cell types could be participating in the remodeling of neuronal circuitry by tagging some disrupted synapses for elimination (stripping) in an attempt to prevent the spread of tissue damage [66,74-78]. Notably, in OHT eyes increased microglial branching was absent in the NFL-GCL, the only retinal layer having rod-like microglia [35]. As described recently by our group, some processes of rod-like microglia penetrate the underlying IPL accompanying the dendrites of ganglion cells [35]. With this observation, it is tempting to postulate that such a relationship would be consistent with the removal or stripping of the disrupted synaptic contacts attributed to rod-like microglia [75,77]. Also, hyper-ramification could increase the microglial area of surveillance, playing a key role in neuronal survival [68-70]. In contralateral eyes, where neuronal damage has not been reported [43], the presence of Iba-1+ cells with an increased level of branching could reflect the presence of tissue stress signals.

### Microglial migration across the retinal parenchyma

Fifteen days after laser treatment, Iba-1+ cells moved to the nearest nuclear layer in both OHT and contralateral eyes. In experimental glaucoma [12,63], as well as in several retinal diseases [13,17,79-87], microglial activation is associated with cell migration which allows microglia to interact and eliminate damaged or dead neurons [61,84,88,89].

Observations in the present study support the idea of the outer retina being impaired by OHT. In contralateral and OHT eyes: i) type 1 OS cell somas migrated closer to the RPE than in the control group. RPE dysfunction has been associated with migration of the resident microglia from the inner retina to the subretinal space in an attempt to support impaired RPE phagocytic functions [90,91]; and ii) type 1 OS cells reoriented their processes from a perpendicular arrangement to one parallel to the retinal surface in both cases. It has been suggested that a characteristic and early feature of microglial activation is their ability to reorient their processes towards the site of injury [66,92]. Notably, this feature was observed in contralateral eyes and the degree of reorientation of processes appeared in an intermediate state between naïve and OHT eyes.

Studies on retinal dystrophies, phototoxicity, and age-related macular degeneration [61,85,87,89,93-97] have demonstrated that chronic activation of microglia could promote a pro-inflammatory environment that would affect RPE morphology and function [98,99]. Activated Iba-1+ cells in the OS could produce pro-inflammatory factors and chemokines capable of inducing blood-cell recruitment [96] that could at least partly explain the presence of rounded Iba-1+ MHC-II+ CD68+ cells in the subretinal space of OHT eyes. Another feature supporting the possibility of the outer retina being affected by OHT is the fact that there were dendritic-like Iba-1+ cells in the OS of OHT eyes in addition to rounded Iba-1+ cells. In the mouse retina, dendritic cells are located in areas of the retina where the retinal barriers could be vulnerable and increased numbers of these cells have been associated with structural abnormalities of the RPE in some mice strains [90]. In our Swiss mice, dendritic-like Iba-1+ cells were restricted to the IPL in naïve and contralateral eyes. By contrast, this cell type was found in the OS in addition to IPL in OHT eyes. Evidence supports the idea of cell communication among retinal microglia, even between microglia in the subretinal space and in the inner retina [87,98]. According to this proposal, the presence of rounded- and dendritic-like Iba-1+ cells in the OS of OHT eyes, two cell types with high antigen-presenting capacity, could relate to outer retinal barrier dysfunction.

It has been reported that chronic tissue stress in glaucomatous eyes may lead to increased contact of the retina and optic nerve head tissues with systemic immune cells due to alterations in perivascular barrier [100]. In the present work, numerous rounded Iba-1+ CD68+ cells were detected in the NFL-GCL of OHT eyes, located mainly adjacent to the major retinal vessels. Iba-1+ rounded cells close to the vessel have been reported in chimeric mice following retinal damage [101]. In addition, it has been postulated that rounded cells could be monocytes entering the retina due to the breakdown into the blood-ocular barrier described in glaucoma [88,100]. In DBA/2J animals transendothelial migration of Iba-1+/CD68+ round cells mediate early damage and their reduction improves neuronal survival [102]. It should be noted that in the contralateral eyes where RGC death has not been reported [43], rounded Iba-1+ CD68+ cells in the NFL-GCL were not found.

### Microglial increased number

Microglial proliferation has been reported in glaucoma [28,30-32,39,42,54,63,103,104]. In patients with glaucoma, microglial proliferation occurs by the expression of growth factors, such as M-CSF and GM-CSF [105], secreted in part by reactive astrocytes [106]. In the same experimental model used here, we have reported astrocyte activation in contralateral and OHT eyes [36]. Such activation could contribute to the significantly increased number of Iba-1+ cells found in contralateral and OHT retinas in the present work. A higher microglial number in glaucoma could be explained by the mitosis of the resident microglia [101,107] or by the entry into the retina of monocytes/macrophages from the bloodstream that later differentiate into microglial cells [14,61,84,90,96,101,107-114]. The mechanism involved in the increased number of microglia observed here is beyond the aim of the present study and deserves further investigation.

### Expression of MHC-II, CD68, Ym1, and CD86

Typically, activated microglial cells are considered to release significant levels of pro-inflammatory molecules, including cytokines and free radicals. Microglia engaged in these responses were also routinely found to exhibit a greater expression of molecules such as CD68, which is a low-density lipoprotein associated with microglial phagocytosis, and MHC-II, in relation to antigen presentation [115]. The expression of MHC-II in glial cells is upregulated in the glaucomatous human retina and optic nerve head [33,36,100,116-118]. Similarly, we found MHC-II upregulation in Iba-1+ cells in contralateral and OHT eyes. In this respect, it was remarkable that no differences between them were detected and that in both instances this activation marker was found throughout all retinal layers. By contrast, the CD68 expression pattern differed between contralateral and OHT eyes in that it was higher, involved more cells, and was distributed in all retinal layers in OHT. Chiu *et al*. [119] reported a correlation between microglia morphology and retinal-ganglion-cell loss in experimental glaucoma. These authors found that fully activated microglia exacerbated RGC loss and that a moderately activated morphology appeared when a neuroprotective agent was given. In the present study, the strongest expression of CD68 in OHT eyes was found in cells displaying features of a fully activated state. CD68 is a member of the scavenger-receptor family. Scavenger receptors typically function to clear cell debris, promote phagocytosis, and mediate the recruitment and activation of macrophages. Thus, the increased expression of CD68 in these OHT eyes seems to be in accordance with the ganglion-cell death reported in this experimental model [43] and the rounded Iba-1+ cells presumably infiltrating the retinal parenchyma (CD68 molecule (*Homo sapiens* (human); Gene ID: 968; provided by RefSeq, Jul 2008).

Positive immunolabeling for Ym1 was restricted to a few ameboid Iba-1+ cells in the NFL-GCL of OHT eyes. To determine microglia polarization was not within the scope of the present study and cannot be based on the results of a single surface marker, however, the fact that only a few cells expressed a surface marker of the M2 phenotype could point towards most Iba-1+ cells in this OHT model exerting functions not related to the M2 activation pattern. Moreover, it should be taken into account that data presented here corresponds to 15 days after lasering and that a transient expression of Ym1, as has been reported during brain ischemic injury in mice [120,121], cannot be ruled out.

As mentioned above, widespread upregulation of MHC-II in Iba-1+ cells took place in all retinal layers in contralateral and OHT eyes, however, in both instances CD86 immunolabeling was restricted to ameboid and rounded Iba-1+ cells in the NFL-GCL and in the OS. CD86- Iba-1+ cells could prevent the functional activation of T cells by their omission of co-stimulation, which may result in T-cell apoptosis or anergy, thus downregulating the immune response [121,122].

### Contralateral eyes: an overview

After tissue injury, reactive microglia are capable of undergoing migratory and proliferative processes through the brain or retina to interact with damaged cells [123]. A noteworthy point of this study was the observation that, in untreated contralateral eyes as well as in OHT eyes, besides the increase in microglia cell number and displacement of these cells across retinal layers, there were additional signs of Iba-1+ cell activation. As in OHT eyes, microglia reactivity affected all retinal layers. It should be mentioned that IOP levels in contralateral eyes did not differ significantly from naïve eyes and that in the same experimental model of OHT used here and at the same time point of the death of mouse, neither RGC death or degeneration nor ERG alteration has been reported [43]. Nevertheless, Iba-1+ cells in contralateral eyes share their greater numbers with OHT eyes, higher level of branching, process shortening and thickening, migration to the nearest nuclear layer, reorientation of processes, smaller arbor area in the plexiform layers, increased retinal area occupied by Iba-1+ cells in the NFL-GCL, and upregulation of MHC-II and CD68 in comparison with naïve eyes. Some of these phenomena are related to a pro-inflammatory environment, synapse disruption, or neuronal damage, among other factors. It has been reported that the degree of microglial activation varies with the severity of neuronal injury and that the mildest injuries may only cause hyper-ramification of microglia [71]. However, as mentioned above, in addition to a widespread higher level of branching, Iba-1+ cells in contralateral eyes exhibited several signs of activation reported in neuroinflammatory diseases, thus reflecting the presence of potentially damaging signals in the tissue that could enhance retinal neural death [46,86,124,125].

## Conclusions

In conclusion, in this study, we show descriptively and quantitatively the differential behavior of activated microglial cells in the different layers of the retina 15 days after unilateral laser-induced OHT. Our data support the notion that, in glaucomatous degeneration, damage extends beyond the GCL and that this is also observed in contralateral untreated eyes. OHT eyes, as well as contralateral eyes, showed several signs of microglial activation, although these were stronger in the former. For this, further dissection of the functional significance of microglial activation in glaucoma onset and progression is mandatory. In addition, contralateral eyes appear to be have potential for discovering points of intervention to which future therapeutics can be directed.

## Abbreviations

GCL: Ganglion cell layer; Iba-1: Ionized calcium binding adaptor molecule 1; Iba1-RA: Area of the retina occupied by Iba-1+ cells; INL: Inner nuclear layer; IOP: Intraocular pressure; IPL: Inner plexiform layer; MHC-II: Major histocompatibility complex class II molecule; NFL-GCL: Nerve fiber layer-ganglion cell layer; OHT: Ocular hypertension; ONL: Outer nuclear layer; OPL: Outer plexiform layer; OS: Photoreceptor outer segment; PB: Phosphate buffer; PL: Plexiform layers; RGC: Retinal ganglion cells; Ym1: also known as CHI3L3 (Chitinase-3-like protein 3) or ECF-L (Eosinophil chemotactic factor-L).

## Competing interests

The authors declare that they have no competing interests.

## Authors’ contributions

FJVS, MAT, MPVP and MVS carried out the development for the animal model and the IOP measurement. AIR, AT, BIG, BR, JMR, JJS, and RdH contributed to immunhistochemical study, analysis and interpretation of data, drafting the manuscript and revising it critically. All authors read and approved the final manuscript.

## Supplementary Material

Additional file 1: Table S1 CD68, CD86, Ym1, and MHC-II expression in Iba-1+ cell types in the retina after 15 days of unilateral laser-induced OHT. Click here for file
