# Supplementary material for: Microglia in mouse retina contralateral to experimental glaucoma exhibit multiple signs of activation in all retinal layers
Source: J Neuroinflammation. 2014 Jul 26;11:133. doi: 10.1186/1742-2094-11-133 (PMC4128533; doi:10.1186/1742-2094-11-133)
Supplement: Additional file 1: Table S1 — CD68, CD86, Ym1, and MHC-II expression in Iba-1+ cell types in the retina after 15 days of unilateral laser-induced OHT. [file 1742-2094-11-133-S1.docx]

**Additional file 1: Table S1. CD68, CD86, Ym1and MHC-II expression in Iba-1+ cell types in the retina after 15 days of unilateral laser-induced OHT.**


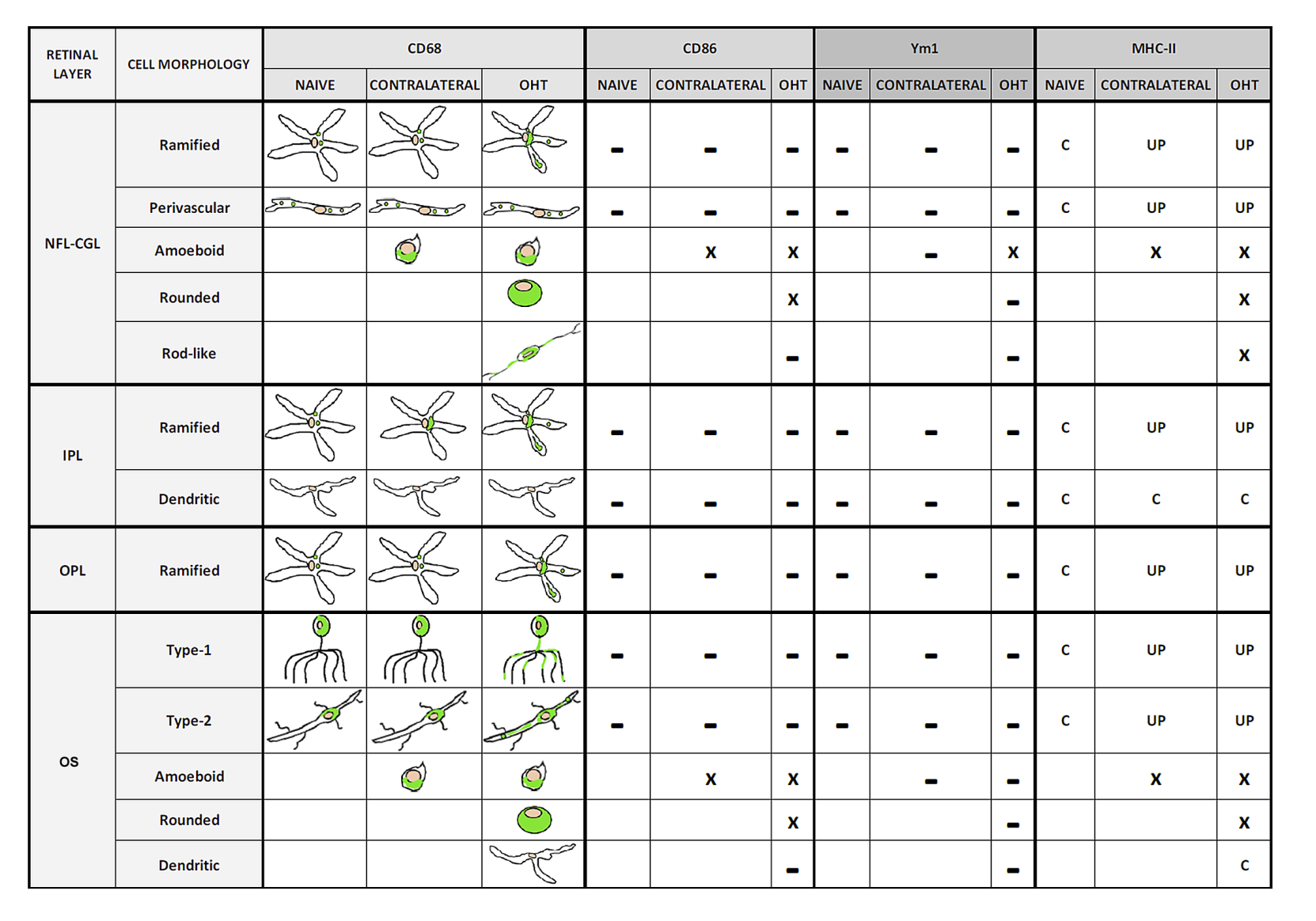


The diagram illustrates staining patterns (green) and expression (yes: x; no: -) of the activation markers in the different morphological types of Iba-1+ cells observed in the retinal layers of naïve eyes, contralateral eyes, and OHT-eyes. (c: constitutive expression; IPL: inner plexiform layer; NFL-GCL: nerve fiber layer-ganglion cell layer; OHT: ocular hypertension; OPL: outer plexiform layer; OS: photoreceptor outer segment; up: upregulated expression).
